# Semi-Supervised Adversarial Learning Using LSTM for Human Activity Recognition

**DOI:** 10.3390/s22134755

**Published:** 2022-06-23

**Authors:** Sung-Hyun Yang, Dong-Gwon Baek, Keshav Thapa

**Affiliations:** Department of Electronic Engineering, Kwangwoon University, Seoul 01897, Korea; shyang@kw.ac.kr (S.-H.Y.); whitedk@kw.ac.kr (D.-G.B.)

**Keywords:** HAR, semi-supervised learning, adversarial learning, syn-LSTM, smart home

## Abstract

The training of Human Activity Recognition (HAR) models requires a substantial amount of labeled data. Unfortunately, despite being trained on enormous datasets, most current models have poor performance rates when evaluated against anonymous data from new users. Furthermore, due to the limits and problems of working with human users, capturing adequate data for each new user is not feasible. This paper presents semi-supervised adversarial learning using the LSTM (Long-short term memory) approach for human activity recognition. This proposed method trains annotated and unannotated data (anonymous data) by adapting the semi-supervised learning paradigms on which adversarial learning capitalizes to improve the learning capabilities in dealing with errors that appear in the process. Moreover, it adapts to the change in human activity routine and new activities, i.e., it does not require prior understanding and historical information. Simultaneously, this method is designed as a temporal interactive model instantiation and shows the capacity to estimate heteroscedastic uncertainty owing to inherent data ambiguity. Our methodology also benefits from multiple parallel input sequential data predicting an output exploiting the synchronized LSTM. The proposed method proved to be the best state-of-the-art method with more than 98% accuracy in implementation utilizing the publicly available datasets collected from the smart home environment facilitated with heterogeneous sensors. This technique is a novel approach for high-level human activity recognition and is likely to be a broad application prospect for HAR.

## 1. Introduction

Human activity recognition has been a concern in Artificial intelligence (AI) research for decades. However, the many proposed approaches face challenges in recognizing human activity accurately and precisely. The HAR system has gained popularity in recent years because of the progress of ubiquitous sensing devices and their capacity to solve specified problems like privacy [1]. HAR systems deployments to the real world in applications such as ambient assisted living (AAL), personal health [2], elderly care [3], defences [4], astronauts [5], and smart homes [6] are potentially increasing. However, there are challenges in the existing techniques to recognize activities substantially since they are now required to account for all unanticipated changes in the real-time scenario.

For example, in this pandemic situation, a COVID-19 patient needs isolation and can be monitored and treated without hospitalization to reduce the burden on isolation centres and hospitals. Sometimes users might modify their schedule of activities without prior knowledge. However, we could anticipate that the system could swiftly understand such new changes; in real-world situations, all these changes are inevitable [7].

Current efforts at HAR focus primarily on detecting changes—finding new activities [8,9] and learning actively—acquiring user annotations about new activities [10]. When a new activity class is added, they must reconstruct and retrain the model from scratch. Some researchers have investigated how an activity model with different activities might develop automatically [11]. This capacity, however, offers the advantage of keeping the knowledge in the business model that has been built through time while lowering training costs, manual configuration and manual feature engineering. Various supervised [12] and semi-supervised [13] methods for activity recognition have been presented. These models provide good accuracy with sufficient data on training. However, their performance from new, undiscovered distributions drops drastically. Therefore, detecting a new user’s activity remains challenging for the model. Most machine learning [14] and deep learning [15] are not conceptually aware of all activities, but they can efficiently recognize human activity with the proper learning and models. The deep neural network is the underlying model for many artificial intelligence models and state-of-art methods. However, deep learning demands a significant amount of data to be a label for learning. Nonetheless, due to practical constraints, some fields of inquiry have data collecting and labeling limitations. As a result, providing enough labeled data in many circumstances is not viable. In the AAL domain, particularly the sensor-based Human Activity Recognition (HAR) problem, data acquisition and labeling tasks necessitate the involvement of human annotators and the use of pervasive and intrusive sensors. Furthermore, the expense of manual annotation, especially for massive datasets, is prohibitively high.

There are two needs for recognizing human activity: improving accuracy, developing trustworthy algorithms to tackle new users, and changing regular activity schedule issues. Therefore, our strategy ensures that the activity identification is addressed mainly through improved performance over previous approaches. This work emphasizes recognition activity by accompanying semi-supervised and adversarial learning on a synchronized LSTM model. To need a system to have the relevant data and ensure that no labels based on the previously learned data can be fully anticipated. Furthermore, this technique could improve performance by utilizing fewer labeling classes. Our method’s highlights are as follows:

- We present semi-supervised and adversarial learning using a synchronized LSTM model to recognize human activity with competitive accuracy.
- The model understands new changes and learns accordingly with reduced error rates; in real-world situations, all these changes are inevitable.
- LSTM is the unsupervised model, but we train it in a semi-supervised feature with a synchronized parallel manner. Therefore, the proposed approach is also an adapted version of LSTM.
- The proposed joint model can structure and learn Spatio-temporal features directly and automatically from the raw sensor data without manual feature extraction. As a result, the model can train unannotated data more easily and conveniently.
- This framework can likely be applied to various recognition domains, platforms, and applications such as natural language processing (NLP), PQRS-detection, fault detection, facial recognition, etc.
- This method could be the best-suited state-of-the-art method for human activity recognition because of its high-level activity recognition ability with reduced errors and increased accuracy.

The proposed method can be used as the external sensor deployment method for a mix of several sensor deployment methods like wearable, external, camera, or all For the user’s convenience. But we evaluated and compared using fully-added real-world data sets collected from external sensors deployed in various corners of the house and apartment from Kasteren and Adaptive System Advanced Studies Center (CASAS). The remaining documents are arranged accordingly. Section 2 describes related work. Section 3 shows our recommended technique. Section 4 provides the experiment set-up, analysis and assessment. Finally, the paper ends in Section 5.

## 2. Related Work

The activity was identified via heterogeneous sensors, wearable sensors, and cameras for ambient assistive living and monitoring [16]. An innovative HAR method uses body-worn sensors that partition the activity into sequences of shared, meaningful and distinguishing states known as Motion Units [17], i.e., a generalized sequence modeling method. However, the external sensor is on researchers’ choice because of body and personal issues [18]. Many approaches that use techniques like deep learning, transfer learning, and adversarial learning are proposed in the state-of-art strategies for HAR. In [19], active learning methodologies for scaling activity recognition apply dynamic k-means clustering. Active learning reduces the labeling effort in the data collecting and classification pipeline. On the other hand, feature extraction is considered a classification problem. [20] evaluates human activities based on unique combinations of interpretable categorical high-level features with applications to classification, learning, cross comparison, combination and analysis of dataset and sensor. Despite all of the improvements made in the suggested model, such as the computational cost reduction, the approaches are still prone to underfitting due to their poor generalization capacity [21]. 

A machine learning Naive Bayes classifier recognizes the most prolonged sensor data sequences [22]. A progressive learning technique dubbed the dynamic Bayesian network has been explored by re-establishing previously learned models to identify activity variation [23]. To extract task-independent feature representations from early generative models, deep learning approaches have been employed on Boltzman machines [24]. More sophisticated models like CNN [25,26] were effectively utilized in complex HAR tasks. Likewise, some suitable methods are employed to categorize certain sorts of activity, such as multilayer perceptrons [27], vector support machine [28], Random forest [29], decision-making tree [30], and an updated HMM [31]. This research aimed to record sensor changes or changes in discrimination models to recognize human activities. Valuable data means data, especially for lesser amounts, which may be employed to generate high performance. These ultimately save on labelling. For this purpose, several techniques are used in the study. Cameras were also used as external HAR sensors. Indeed, significant research has identified activities and actions in video sequences [32,33]. The mentioned work is particularly suited for safety applications and interactive applications. However, video sequences have particular problems with HAR, privacy and pervasiveness.

Adversarial machine learning has gained increasing interest with the advent of Generative Adversarial Networks (GANs) [34], and it now achieves excellent performance in a wide range of fields, including medicine [35,36], text and image processing [37,38], and architecture [39,40]. GANs work by pitting generator and discriminator algorithms against one another in order to distinguish between produced and real-world data. Deep learning is used to create discriminators that continually learn the best set of features, making it difficult for the generator to pass the discriminator test [41]. The difficulty of providing synthetic data was addressed in the first attempts to use adversarial machine learning for HAR. However, improving categorization algorithms remains the most pressing issue in this sector.

It is challenging to obtain labelled data from users for practical applications. However, unlabeled data can be collected. Since semi-supervised learning uses both the labelled and unlabeled data for model training, the respective models can capture the characteristics of unlabeled data left-out users and further enhance validation performance. Furthermore, adversarial semi-supervised learning models compete with a state-of-the-art method for many areas, such as the classification of images [42] and material recognition [43]. Therefore, the adversarial semi-supervised [44] model is a viable solution. However, unlike other semi-supervised learning techniques, adversarial semi-supervised learning methods are generally applied to circumstances in which unlabeled data is available [45,46].

## 3. Proposed Method

**Figure 1:**
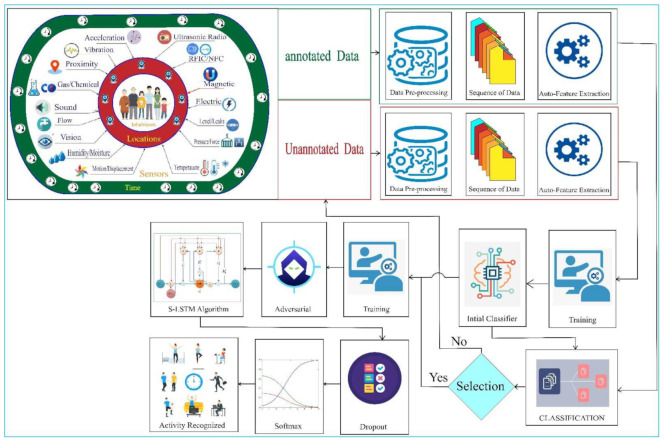
System workflow of our proposed method for HAR.

Human activity recognition systems consist of data acquisition, pre-processing, feature extraction and training/testing phases. Our approach also contains the same process, but the driving factor is new in HAR. The workflow of our proposed method is shown in Figure 1. Heterogeneous sensors were deployed in the apartment’s different locations. The data from the sensors are pre-processed by doing segmentation and filtrations. As we use the deep learning model, the feature is extracted automatically. Then, we train and classify the activity and recognize it. If data is unannotated, we reprocessed it and classified it. Finally, we add some perpetuation to develop the self-immune system to the network as an adversarial learning mechanism. We can benefit from training the unlabeled data and labeled data. Similarly, it minimized the error by adversarial learning techniques that can boost the accuracy of the HAR. Hence, we present the Semi-supervised adversarial learning mechanism to detect the human activity facilitated by the synchronized LSTM model that is novel in HAR to this date. 

### 3.1. Semi-Supervised Learning

Supervised learning [47] is a strategy employed by learning data and labels in many domains or environments. Supervised learning knows and uses labelled data and is helpful for large-scale issues. Various machine learning and deep learning approaches have been used as the supervised learning mechanism. However, hundreds to millions of learning data can be provided to train, and labelling each data is vital. Therefore, supervised learning cannot be used without sufficient learning data because of these issues. Semi-supervised learning is a mechanism to address these deficiencies [48]. It is a technique used to recognize unlisted data with essential criteria like thresholds and re-learn models using available learning data to increase performance based on the projected values of the learned sequences. The semi-supervised method reduces manual annotation and helps develop a self-learning model, which gains robust knowledge and eventually increases the recognition efficiency or accuracy of the recognition model. The feedback properties of LSTM are used to send the unannotated. Then the unannotated data is trained and annotated.

### 3.2. Sync LSTM

**Figure 2:**
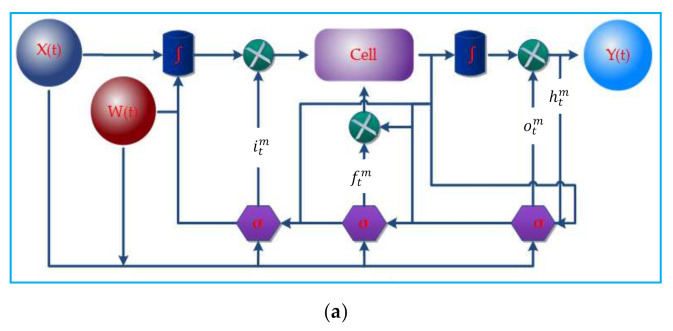

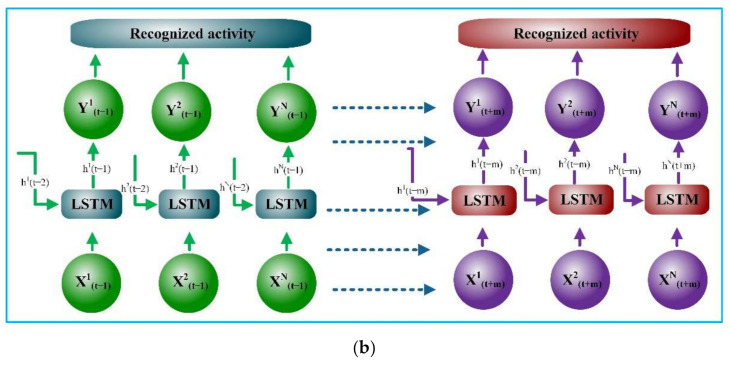
(**a**) Internal Architecture of sync-LSTM; (**b**) Unfold of sync-LSTM.

Sync LSTM is the adapted LSTM based on artificial recurrent networks (RNN). The insight of the LSTM and unfolded sync-LSTM network is shown in Figure 2a,b, respectively. It can handle multiple data streams at a time [49]. A conventional LSTM neuron takes a lengthy time to process a signal with significant time steps. As a result, we simultaneously deployed numerous LSTM units to process different data streams. The input streams of data are vectored as $x\in \mathbb{R}\left(I\times F\times S\times V\times P\right)$ in which *I* and *F* are the initial and final end times. Similarly, *S* denotes the sensor ID, *V* is the sensor’s data value, and *P* represents the sensor location. $x_{t}^{m}=\left(x_{t}^{1},x_{t}^{2},x_{t}^{3}\ldots .x_{t}^{N}\right)$ is Sync-LSTM sample inputs where each data is a individual set *m* = 1, 2, 3, … *N* sampled at time *t* = 1, 2, 3, … *N*−1. The input data vector b$x_{t}^{1},\;x_{t}^{2}\ldots x_{t}^{N}\in \mathbb{R}\;\left[\left(S_{1}\times E_{1}\times I_{1}\times V_{1}\times L_{1}\right),\;(E_{2}\times I_{2}\times V_{2}\times L_{2}\right),\;\ldots (S_{N}\times E_{N}\times I_{N}\times V_{N}\times L_{N})]$. $Y_{t}^{1},\;Y_{t}^{2},\;Y_{t}^{3}\ldots Y_{t}^{N}$ resembles the output through the hidden states $h_{t}^{1},h_{t}^{2},\;h_{t}^{3}\ldots \ldots h_{t}^{N}\;$at the time *t*.

(1) $$i_{t}^{m}=\sigma \left(w_{xi}\times x_{t}^{m}+w_{hi}\times h_{t-1}^{m}+w_{ci}\times c_{t-1}^{m}+b_{i}\right)$$

(2) $$f_{t}^{m}=\sigma \left(w_{xf}\times x_{t}^{m}+w_{hf}\times h_{t-1}^{m}+w_{cf}\times c_{t-1}^{m}+b_{f}\right)$$

(3) $$o_{t}^{m}=\sigma \left(w_{xo}\times x_{t}^{m}+w_{ho}\times h_{t-1}^{m}+w_{co}\times c_{t-1}^{m}+b_{o}\right)$$

(4) $$c_{t}^{m}=f_{t}^{m}\times c_{t-1}^{m}+i_{t}^{m}\times tanh\times \left(w_{xc}x_{t}^{m}+w_{hc}h_{t-1}^{m}+b_{c}\right)$$

(5) $$h_{t}^{m}=o_{t}^{m}\times tanh\times c_{t}^{m}$$

(6) $$h_{t}^{m}=H\left(w_{x^{m}h^{m}}\times x_{t}^{m}+w_{h^{m}h^{m}}\times h_{t-1}^{m}+b_{h}^{m}\right)$$

(7) $$Y_{t}^{m}=\left(w_{y^{m}h^{m}}\times h_{t-1}^{m}+b_{y}^{m}\right)$$

H is the composite function, where the $i_{t}^{m};$ input gate, $f_{t}^{m};$ the forget gate, $o_{t}^{m};$ the output gate and the $c_{t}^{m};$ cell memory with W(t); weight matrix. Every given gate has its activation functions σ; sigmoid and ∫; hyperbolic tangent. 

It comprises an input layer, the LSTM parallel layers, and the outputs wholly linked. In the last stage, the result is summed up as n × h, where h is the number of neurons buried in each LSTM unit. After each step, the LSTM layers update their inner state. Finally, the weight, bais, and hidden layers are allocated to 128. The number of classes determines the final number of parameters.

### 3.3. Adversarial Training

**Figure 3:**
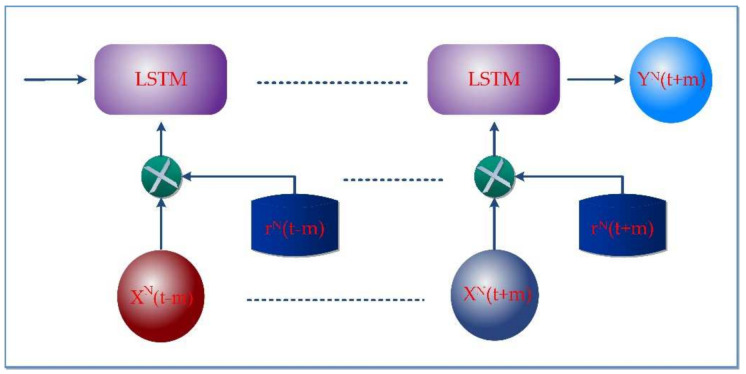
Adding of Adversarial Function.

Adversarial learning is a technique to regularize neural networks that improve the prediction performance of the neural network or approaches to deep learning by adding tiny disturbances or noises with training data that increases the loss of a more profound learning model. The schematic diagram of the adversarial learning is shown in Figure 3. However, it proposed that small perturbations to deep learning input may result in incorrect decisions with high confidence [50]. If x and θ are the input and different parameters for a predictive model, adversarial learning adds the following terms to its cost function in the training phase to classify the correct class.

(8) $$\log p\;(Y_{t}^{m}|X_{t}^{m}+r_{t}^{m};\theta )=where\;r_{t}^{m}=arg\min\log p\;(Y_{t}^{m}|X_{t}^{m}+r_{t}^{m};\hat{\theta })$$

From Equation (8), r is adversarial in the input data. $\hat{\theta }$, is a set of the constant parameter of the recognition model. At each training, the proposed algorithm identifies the worst-case perturbations $r_{t}^{m}$ Against the currently trained model to θ. Contrary to other techniques of regularization, such as dropout, that add random noise, adversarial training creates disturbances or random noise that may readily be misclassified in the learning model by changing input instances. 

Algorithm 1 represents the detailed steps of the recognition system, adding the adversarial function. The adversarial function is a small perturbation that maximizes the loss function. As a result, the predictive accuracy or predictive model is eventually improved by reducing the cumulative loss function of the predictive models.
**Algorithm 1** Sync-LSTM Model with Adversarial Training*Step 1. initialize network* *Step 2. reset: inputs = 0, activations = 0* *Step 3. initialize the inputs* *Step 4. Create forward and backward sync-LSTM* *Calculate the gate values:* *input gates:*$i_{t}^{m}$ *forget gates:*$f_{t}^{m}$ *loop over the cells in the block* *output gates:*$o_{t}^{m}$ *update the cell:*$c_{t}^{m}$ *final hidden state/* $h_{t}^{m}=H\left(w_{x^{m}h^{m}}x_{t}^{m}+w_{h^{m}h^{m}}h_{t-1}^{m}+b_{h}^{m}\right)$ *final output:*$Y_{t}^{m}=\left(w_{y^{m}h^{m}}h_{t-1}^{m}+b_{y}^{m}\right)$ *Step 5. Predict and calculate the loss function* *Calculate seq2seq loss* *Calculate class loss using cross-entropy* *Step 6. Add random perturbations,* $\log p\;(Y_{t}^{m}|X_{t}^{m}+r_{t}^{m};\theta )=where\;r_{t}^{m}=arg\min\log p\;(Y_{t}^{m}|X_{t}^{m}+r_{t}^{m};\hat{\theta })$ *Step 7. Calculate loss function by adding adversarial loss* *Step 8. Optimize the model based on AdamOptimizer*

Algorithm 2 presents a semi-supervised learning framework that guides how unannotated from multiple inputs can be incorporated into a sync-LSTM recognition model.
**Algorithm 2** Semi-Supervised Sync-LSTM Model*Step 1. Recognize unlabeled data based on Algorithm 1* *Step 2. Add recognized dataset to original training dataset* *Step 3. Retrain the model using Algorithm 2*

## 4. Experimental Configurations and Parameters

The detailed results in both the training and recognition are presented in this section. First, several design hypotheses are assigned and processed. Then, the proposed model is trained with the labelled and unlabeled data, and the results are compared with the existing model outputs. Finally, Milan, Aruba, and the House-C datasets are considered for the experimental analysis of the proposed approach from the CASAS dataset and Kasteren house.

### 4.1. Datasets

**Figure 4:**
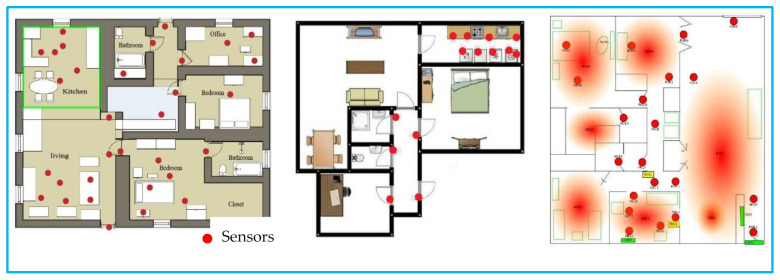
Floor Plan and Sensor Deployment.

The Kasteren dataset [51] and CASAS dataset [52] from WSU have been used to evaluate our proposed method. Table 1 shows an overview of the datasets. The data was collected in an apartment with more than five rooms. In Milan, 33 sensors are installed, whereas in house-C, 21 sensors are installed, and in Aruba, 34 sensors are installed. For the Milan dataset, motion, door, and temperature sensors are the primary sources of sensor events. A woman and a dog were the primary annotated resident in the Milan dataset. The woman’s children occasionally visited the house as an unlabeled resident. The seventy-two days were spent collecting the data from the Milan house. A total of fifteen activities are recorded as the annotated data. One resident in the House-C dataset performed sixteen different activities for twenty days. The sensors show the change of state according to the occupant’s action. The data for the Kasteren House-C is recorded using radiofrequency identification, a wireless sensor network, mercury contacts, a passive infrared-PIR, float sensors, a pressure sensor, a reed switch, and temperature sensors. CASAS Aruba dataset is another trademark dataset that collected eleven annotated activities for two hundred and twenty-two days. The schematic layout of the sensor deployment is shown in Figure 4.

### 4.2. Parameter Setting and Training

The proposed method is trained and tested using the TensorFlow_GPU1.13.1 library and scikit-learn. The obtained data is pre-processed and sampled in overlapping sliding windows with a fixed width of 200 ms with a window length ranging from 0.25 s to 7 s. Our algorithm is examined using an i7 CPU topped with 16 GB RAM and GTX Titan GPU processed on CUDA 9.0 using the cuDDN 7.0 library. The CPU and GPU are employed to minimize the amount of memory used. The dataset is divided into three sections: a training set, validation, and testing. 70% of data is used for training, 10% for validation, and the rest for testing. The data is validated using the k-fold CV (cross-validation). We used 10-fold cross-validation (K = 10) to confirm our findings. The outcome of the accuracy test is averaged, and the error is determined as follows.

(9) $$CV=\frac{1}{p}\overset{10}{\underset{p=1}{\sum }}E\;\left(error\right)\;$$

The dropout rate is adjusted to 0.5 during training to eliminate unnecessary neurons from each hidden layer to alleviate overfitting. Random initialization and training parameter optimization can also help to reduce training loss. To avoid overfitting and make the model stable, cross-entropy and L2 normalization are incorporated.

(10) $$L=-\frac{1}{k}\overset{n}{\underset{k=1}{\sum }}y_{t}^{m}.logy_{t}^{m^{\prime }}+\Gamma .W,$$

In Equation (10), *k* is the number of samples per batch and w denotes the weighting parameter. $y_{t}^{m}$ is the recognized output, and $y_{t}^{m^{\prime }}$; the label. L2 normalization reduces the size of weighting parameters, preventing overfitting. Adversarial learning is a technique for regularizing neural networks. It also improves the neural network’s prediction performance. It perhaps approaches deep learning by adding tiny disturbances or noises to the network with training data that increases the loss of a more profound learning model for regularization, improving recognition ability as adversarial training. If $r_{t}^{m}$ is the adversarial input, then is θ the perturbations, which is written as

$$=arg\min\log p\;(Y_{t}^{m}|X_{t}^{m}+r_{t}^{m};\hat{\theta })\;$$

We strive to tune the optimum hyperparameters, as indicated in Table 2, so that the learning rate and L2 weight decrease and the difference decreases, resulting in the most significant possible performance. For the Milan, House-C, and Aruba datasets, we employ a learning rate of 0.005, 0.004, and 0.006 and a batch value of 100 for each epoch to train the model. For all sets, learning begins at 0.001. The training lasts roughly 12,000 epochs and ends when the outputs are steady. The Adam optimizer is an adaptive moment estimator that generates parameter-independent adaptive learning rates. The input dimension is set to 128, and the output dimension is set to 256. The gradient crossover threshold is reduced by adjusting gradient clipping to 5, 4, and 5. 

### 4.3. Evaluation Parameters

Accuracy, F1-score, and training time evaluate the model’s performance. These can be calculated using the confusion matrix, where the row represents the predicted class, and the column represents the actual class. Human activity recognition is evaluated according to their computational recognition accuracy, resulting from the Precision and Recall parameters. Precision is the proportion of correctly recognized instances from perceived activity occurrences. A recall is the proportion of correctly identified instances out of the total events. The f-score is the weighted average of Precision and Recall between 0 and 1. The better performance indicated if closer to 1

(11) $$Precision=\frac{tp}{tp+fp\;}\times 100$$

(12) $$Recall=\frac{tp}{tp+fn\;}\times 100$$

(13) $$Accuracy=\frac{tp+tn}{tp+tn+fp+fn\;}\times 100$$

(14) $$f-score=\frac{2\times Precision\times Recall}{Precision+Recall\;}$$

where tp; true-positive, tn; true-negative, fp; false-positive, and fn; false-negative. The tp is the number of true activities detected in positive instances, while an  fp indicates the false activities detected in negative instances. The fn score indicates the exact number of false activities detected in positive instances, whereas the tn score reflects the correct non-detection of activities in negative instances. 

## 5. Results and Evaluations

### 5.1. Recognition Analysis

The activity is recognized according to the proposed smart home development method. The method shows a tremendous recognition result. Table 3 shows the confusion matrix of Milan, showing the correctly recognized instances out of the perceived activity occurrences and correctly recognized instances out of the total occurrences. Thus, all the activities have more than 95% of recognition accuracy to the given instances. According to the confusion matrix, the *Bed-to-Toilet* activity was correctly detected with 95% accuracy but still has an activity error of 5%. The *Bed-to-Toilet* may create confusion with *Sleep* activity and *Morning_Meds* these activities are very closely related. Fortunately, *Eve_Meds* has a 100% confusion accuracy. The activities *Chores, Desk_Activity, Dining_RM_Activity, Guest_Bathroom, Kitchen_Activity, Leave_Home, Master_Bathroom, Mediate, Watch_TV, Sleep, Read,* and *Master_Bedroom_Activity* recognition accuracies of 98%, 98%, 99%, 97%, 97%, 96%, 99%, 98%, 99%, 97%, 96%, 95% and 95%, respectively. Although the obtained result is good enough, we still struggle to get the 100%, letting some errors. The errors arise because of confusion between similar activities, activities performed in the same room, and the different activities performed simultaneously with the same instances. Sometimes we performed the same activities with different people, which was unannotated.

In this dataset, the house owner’s daughter often visited her house, performed the same task, and was recognized more accurately. The confusion matrix for House-C is shown in Table 4. The number of activity instances is relatively few, so errors are relatively low, and recognizing the activity with true positive value results in 98.01% accuracy. 

House-C has achieved 98.11% precision, 98.109% recall, and 0.98 f1-score. Activities such as *brushing teeth* (95% accurate), *Showering* (97%), *Shaving* (95%) *toileting* (93%) create confusion and errors because all the activities happen in one location. However, the errors that occur are comparatively low, so that they can be neglected. Furthermore, *Preparing Breakfast* (97%), *Preparing Lunch* (96%), *Preparing Dinner* (98%), *Snacks* (97%) and *Eating* (99%) have good recognition accuracy but still have some errors because of confusion among these activities as they share some sensor values. House-C’s dataset is insufficient to establish the experimental concept fully and has 100% recognition accuracy. Although the accuracy is good, more data and training could be needed to find actual recognition.

Nevertheless, we confirm that our proposed approach for human activity recognition is feasible. In Aruba, the number of instances per activity type varies considerably as shown in the Table 5. The proposed system allows most activities to be recognized with an overall accuracy of 98.34% and an F1-score of 0.98. However, some activities have 100% accuracy and some have less recognition accuracy, such as Enter Home of 95%. The *Enter_House* and *Leave_House* activities involve the same main door and sensors, taking their occurrences into account. Likewise, *Wash_Dishes* gets mistaken with *Meal_Preparation* since both are done in the kitchen, sharing the same occurrences. The *Wash_Dishes* action may also be performed during *Meal_Preparation* and can therefore be regarded as concurrent.

### 5.2. Recognition Comparison

**Figure 5:**
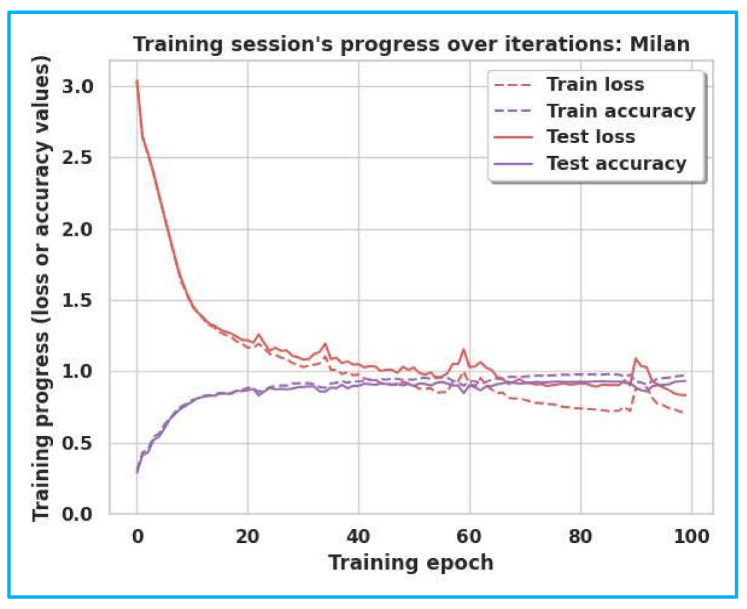
Training/Test Accuracy/Loss for Milan.

**Figure 6:**
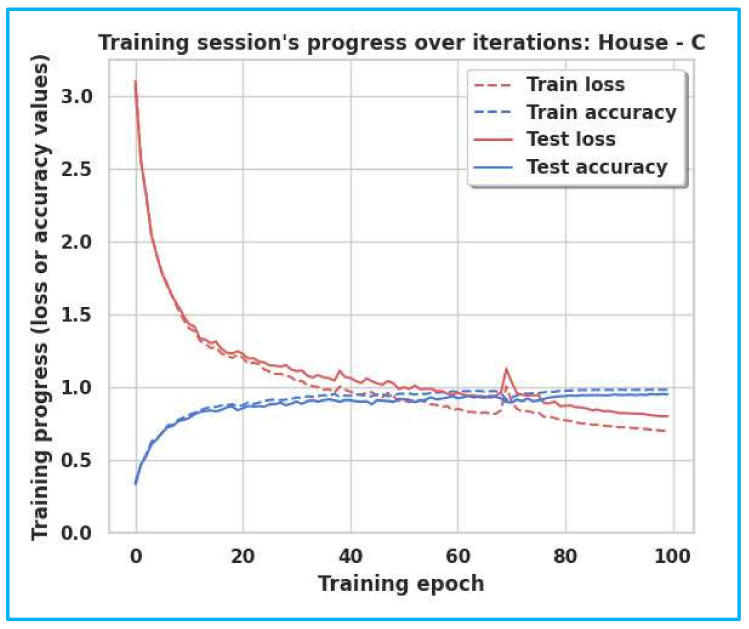
Training/Test Accuracy/Loss for House-C.

**Figure 7:**
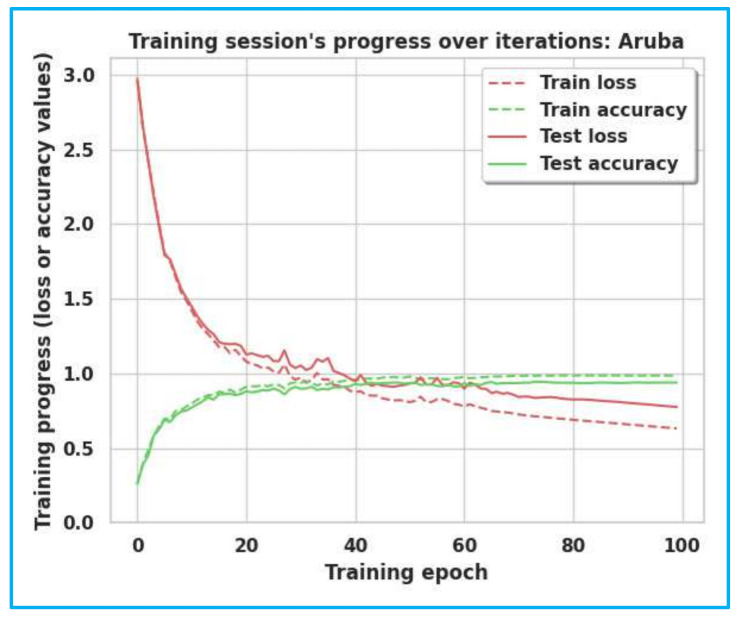
Training/Test Accuracy/Loss for Aruba.

The accuracy and loss curves of Milan, House-C, and Aruba are shown in Figure 5, Figure 6 and Figure 7. The gap between the training and testing accuracy in the graphs is comparatively tiny, indicating the model’s effectiveness. Furthermore, the gap between training and test loss is very narrow, explaining that dropout techniques, adversarial training, and semi-supervised learning are beneficial. 

**Figure 8:**
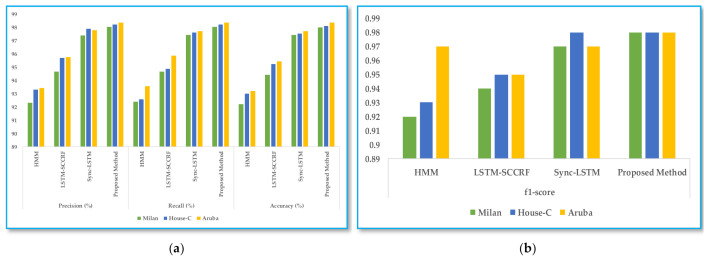
(**a**) the average precision, recall, and accuracy and (**b**) the f1-score comparison with different models.

The average accuracy was 98.154%, and the average error was 0.1571. The performance result of the proposed approach with the existing framework, such as HMM, LSTM, and sync-LSTM methods (algorithms), is based on average precision, recall, and accuracy, as shown in Figure 8a and f-score in Figure 8b. The accuracy of our work is more than 98%, and the f1-score is more than 0.98. The sync-LSTM also has competitive accuracy with our method but cannot deal with new or unannotated data. The analysis reveals that the presentation method can be more accurate than the current approaches.

## 6. Conclusions

The presented work in this paper shows that semi-supervised adversarial sync-LSTM can produce a feasible solution for detecting human activities in the intelligent home scenario—a comprehensive comparison with recently introduced activity recognition techniques, such as HMM, LSTM, and sync-LSTM. LSTM can work with single data sequences, and sync-LSTM can accept multiple inputs and generate synchronized and parallel outputs. Still, these techniques fail to address the new and unknown data in the sequence. Many approaches have been researched on annotated and regular activity detection. However, few of them have tried to detect complex and unannotated activity. The proposed method used the improvised LSTM and its semi-supervised learning ability to recognize complex and unannotated human activity from the data collected from the sensors in the smart home. The adversarial learning technique increases learning ability by adding tiny disturbances or noises to the network. Accuracy, processing complexity, complex activity and unannotated activity recognition are still challenging issues in human activity recognition. However, the precision and recall are also excellent, resulting in an f1 score of more than 0.98 and 98% accuracy.

Nevertheless, the accuracy is not equal or tends to be one hundred percent due to shared location, sensor timing, noise interference, and limited data. The existing best-performing model faces several real-time challenges while dealing with different datasets. The number of activities performed, sensor types, sensor deployment, number of inhabitants, and periods are vital parameters affecting model performance. In addition, the window size also plays a crucial role in model performance because small windows may not contain all the information, and large windows may lead to overfitting and overload. Recognizing the unannotated data and processing it in parallel is beneficial for highly imbalanced datasets. 

The computational complexity is O(W), where W is the weight and relies on the number of weights. The weight is determined by the number of output units, cell storage units, memory capacity, and hidden unit count. The amount of units connected with forwarding neurons, memory cells, gate units, and hidden units also impacts. The length of the input sequence has no bearing on the computational complexity. Using an LSTM framework for the labelled and unlabeled data adds time complexity, yet our method has a reasonable calculation time of 9 s.

Besides detecting unannotated activity, the proposed method can automatically extract Spatio-temporal information by consuming less pre-processing time and manual feature extraction. In addition, external sensors were used instead of body-worn and video sensors to protect the user’s privacy and avoid body hurdles. Furthermore, more complex, multi-user, and multi- variants activities can be recognized by enhancing and upgrading the proposed method in the future. Moreover, we can take advantage of cloud computing, edge computing and IoT services to process a large amount of data for better performance. Finally, our approach can be used in other domains and environments like sign language detection, cognitive abilities, etc. Hence, our suggested approach is a better state-of-art method for HAR. 

## Figures and Tables

**Table 1:** Outlines of Datasets.

Description	Milan	House-C	Aruba
Setting	Apartment	House	Apartment
Rooms	6	6	5
Senors	33	21	34
Activities	15	16	11
Residents	1	1	1
period	72 days	20 days	220 days
Activities Performed	Bed-to-Toilet, Chores, Dining_Rm_Activity, Eve_meds, Guest_Bathroom, Kitchen_Activity, Leave_Home, Master_Bathroom, Meditate, Watch_TV, Sleep, Read, Morning_Meds, Master_Bedroom_Activity	Brushing teeth, Drinking, Dressing, Eating, Leaving House, Medication, Others, Preparing Breakfast, Preparing Lunch, Preparing Dinner, Relax, Sleeping, Showering, Snack, Shaving, Toileting	Meal_Preparation, Relax, Eating, Work, Sleeping, Wash_Dishes, Bed_to_Toilet, Enter Home, Leave Home, Housekeeping, Resperate

**Table 2:** Hyperparameter Configurations.

Hyperparameters	Values		
	Milan	House-C	Aruba
Time Steps of input	128	128	128
Initial Learning Rate	0.001	0.001	0.001
Learning Rates	0.005	0.004	0.006
Momentum	0.5	0.5	0.5
Optimizer (Bi-LSTM)	Adam	Adam	Adam
Batch Size	100	100	100
Dropout Rate	0.5	0.5	0.5
Batch Size	100	100	100
Epochs	12,000	12,000	12,000

**Table 3:** Confusion matrix for Milan dataset.

Activity		1	2	3	4	5	6	7	8	9	10	11	12	13	14	15	Recall
1	**Bed-to-Toilet**	95	0	0	0	0	0.3	0	0	0	0	0	0	0	1	0.3	98.344
2	**Chores**	0	98	0	0	0	0	1	0.2	0.05	0	0	0	0	0	0	98.741
3	**Desk_Activity**	0	0	98	0	0	0	0	0	0	0	2	0.3	0	0	0	97.707
4	**Dining_Rm_Activity**	0	0.8	0	99	0	2	0	0.1	0	0	0	0	0	0	0	97.154
5	**Eve_Meds**	0	0	0	0	100	0	0	0	0	0	0	0	0	0	0	100.000
6	**Guest_Bathroom**	0	0	0	0.3	0.2	97	0	1.2	1	0	0	0	0	0	0	97.292
7	**Kitchen_Activity**	0	1.2	0	1.2	0	0	97	0	0.3	0	0	0	0.6	0	0	96.710
8	**Leave_Home**	0	0	0	2	0	0	1.2	96	0.9	0	0	0	1	0	0	94.955
9	**Master_Bathroom**	0	1.1	0	0	0	0	1	0.4	99	0	0	0	0.2	0	0	97.345
10	**Meditate**	0	0	0	0	0.3	0	0	0	0	98	0.6	0	0	0	0	99.090
11	**Watch_TV**	0	0	0	0	0	0	0	0	0	0	99	0	0	0	0	100.000
12	**Sleep**	2	0	0	0	0	0	0	0	0	0	0	97	0	1	0.5	96.517
13	**Read**	0	0	0	1	0	1	0	0	0	0	0	0	96	0	0.9	97.068
14	**Morning_Meds**	1	0	0.5	0	0	0	0.8	0	0	0	0	0	0	95	0.033	97.603
15	**Master_Bedroom_Activity**	0	0	0	0	0	0	0	0	0	0	0	0	0	0.2	95	99.790
	**Precision**	96.939	96.934	99.492	95.652	99.502	96.710	96.040	98.059	97.778	100.000	97.441	99.692	98.160	97.737	98.208	

**Table 4:** Confusion matrix for House-C.

Activity		1	2	3	4	5	6	7	8	9	10	11	12	13	14	15	16	Recall
1	**Brushing Teeth**	95	0	0	0	0	0.3	0	0	0	0	0	0	0	0.3	0.3	0.5	98.548
2	**Drinking**	0	98	0	0	0	0	1	0.2	0.05	0	0	0	0	0	0	0.2	98.542
3	**Dressing**	0	0	98	0	0	0	0	0	0	0	0.5	0.3	0	0	0	0.1	99.090
4	**Eating**	0	0.3	0	99	0	2	0	0.1	0	0	0	0	0	0	0	0.05	97.585
5	**Leaving House**	0	0	0	0	100	0	0	0	0	0	0	0	0	0	0	0.1	99.900
6	**Medication**	0	0	0	0.3	0.2	97	0	0.2	1	0	0	0	0	0	0	0.4	97.881
7	**Preparing Breakfast**	0	0.2	0	0.2	0	0	97	0	0.3	0	0	0	0.3	0	0	0.5	98.477
8	**Preparing Lunch**	0	0	0	2	0	0	1.2	96	0.9	0	0	0	0.5	0	0	0.1	95.333
9	**Preparing Dinner**	0	0.1	0	0	0	0	1	0.4	99	0	0	0	0.2	0	0	0.3	98.020
10	**Relax**	0	0	0	0	0.3	0	0	0	0	98	0.6	0	0	0	0	0.5	98.592
11	**Sleeping**	0	0	0	0	0	0	0	0	0	0	99	0	0	0	0	0.43	99.568
12	**Showering**	1	0	0	0	0	0	0	0	0	0	0	97	0	1	0.5	0.2	97.292
13	**Snacks**	0	0	0	1	0	1	0	0	0	0	0	0	96	0	0.4	0.1	97.462
14	**Shaving**	1	0	0.5	0	0	0	0.8	0	0	0		0	0	95	0.033	0.4	97.204
15	**Toileting**	0	0	0	0	0	0	0	0	0	0	0	0	0	0.2	93	0.11	99.668
16	**Others**	0.2	0	0.1	0.2	0.3	0.2	0.3	0.11	0.2	0.1	0	0.58	0.3	0.5	0.2	93	96.583
	**Presicion**	97.737	99.391	99.391	96.397	99.206	96.517	95.755	98.959	97.585	99.898	98.901	99.101	98.664	97.938	98.483	95.886	

**Table 5:** Confusion matrix for Aruba.

Activities		1	2	3	4	5	6	7	8	9	10	11	Recall
1	**Meal_Preparation**	98	1.3	0.7	0	0	1.1	0	0	0	0	0	96.934
2	**Relax**	0	98	0	1	1	0	0.3	0	0	0	0.1	97.610
3	**Eating**	0	0	97	0	0	1	0	0	0.5	0.1	0	98.377
4	**Work**	0.6	1.2	0.2	95	0.1	0.6	0.4	1	0.3	0	0	95.573
5	**Sleeping**	0	0	0	0	97	0	0	1	0	0	0	98.980
6	**Wash_Dishes**	0	0	0	0.3	0.2	99	0	0	0	0	0	99.497
7	**Bed_to_Toilet**	0	0	0	0	0	0	98	1	0	0	0	98.990
8	**Enter_Home**	0	0.4	0	0	2	0	1.54	98	0	0	0	96.135
9	**Leave_Home**	0	0	0	0	0	0	0	0	100	0	0	100.000
10	**Housekeeping**	0.2	0	0	0	0	0	0	0	0	98	0	99.796
11	**Resperate**	0	0	0	0	0	0	0	0	0	0	97	100.000
	**Precision**	99.190	97.126	99.081	98.650	96.710	97.345	97.765	97.030	99.206	99.898	99.897	
